# Oil–Water Flow Monitoring in Wellbores with Inflow Control Valves Using Distributed Acoustic Sensing

**DOI:** 10.3390/s26123729

**Published:** 2026-06-11

**Authors:** Chuang Xiao, Ge Jin, Yilin Fan

**Affiliations:** 1 Geophysics Department, Colorado School of Mines, Golden, CO 80401, USA; gjin@mines.edu; 2 Petroleum Engineering Department, Colorado School of Mines, Golden, CO 80401, USA; yilinfan@mines.edu

**Keywords:** multiphase flow, ICV, distributed fiber-optic sensing, production monitoring

## Abstract

Intelligent completion technologies, including Inflow Control Valves (ICVs), have become increasingly important for remotely managing zonal production in complex well architectures. However, quantifying flow rates and phase fractions in such systems remains challenging due to space constraints and the harsh downhole environment, which limit the deployment of conventional sensors. Distributed Acoustic Sensing (DAS) provides a promising solution by converting standard fiber-optic cables into dense arrays of acoustic sensors. While DAS has been successfully applied in applications such as integrity monitoring and leak detection, its use for direct two-phase flow characterization within intelligent completions remains largely unexplored. In this study, we present a DAS-based methodology to monitor and analyze oil–water two-phase flow in horizontal experiments that mimic field conditions. Acoustic data collected from DAS are transformed into time–frequency spectrograms using Short-Time Fourier Transform (STFT) to extract dynamic spectral features. These features are then correlated with pressure drop across the ICV and flow rate, revealing distinct frequency band behaviors associated with fluid changes. To quantify flow characteristics, a power-law model is trained using spectral features to predict flow rate and phase fractions. The results demonstrate strong predictive capability for pressure drop and flow rate under controlled laboratory conditions, highlighting the potential of DAS for multiphase flow diagnostics in field applications with intelligent completions, while water cut prediction remains challenging due to the complex and non-unique relationship between flow conditions and DAS response and is left for future work. This research not only provides new insights into the acoustic response of oil–water flows but also introduces a data-driven framework for leveraging DAS in real-time flow monitoring and control within ICV-equipped completions.

## 1. Introduction

Two-phase oil–water flow commonly occurs in various industrial systems, particularly in those associated with petroleum, such as production systems and transportation pipelines, as reservoirs mature and enhanced oil recovery methods are applied. Accurate monitoring of oil–water flow in production wells is critical for optimizing reservoir performance, improving recovery efficiency, and ensuring flow assurance [1]. In horizontal wells, the behavior of oil–water flow can vary significantly along the wellbore due to gravity segregation, uneven production contributions from different zones, pressure differences, and variations in fluid properties. Without reliable real-time flow measurements, operators may miss early signs of water breakthrough, flow instability, or inefficient production, all of which can lead to economic losses and operational challenges [2]. These challenges have driven the development of advanced completion strategies that allow not only improved flow control but also robust, distributed sensing solutions.

Inflow Control Valves (ICVs) are a critical component of intelligent well completion systems, playing a central role in optimizing reservoir management and enhancing production efficiency [3]. These valves are remotely operated from the surface and are typically installed at strategic intervals along horizontal or multilateral wellbores to regulate inflow from different reservoir zones [4]. By adjusting the valve openings, operators can control fluid contributions from each zone, helping to mitigate unwanted water or gas production and to delay water or gas breakthroughs, thereby improving overall recovery and reducing operating costs. In production wells, ICVs enable selective choking of high water cut zones by adjusting the sleeve position to restrict or shut off inflow from inefficient segments of the reservoir. This zonal control enhances sweep efficiency and supports more uniform reservoir depletion. Unlike autonomous inflow control devices (AICDs), which passively respond to fluid properties, ICVs allow active surface control, offering more flexibility for dynamic production optimization [5]. Despite their benefits, effective utilization of ICVs demands accurate monitoring of multiphase flow behavior across the controlled zones. However, downhole flow measurements remain challenging due to limitations in sensor placement, harsh operating conditions, and the complex nature of multiphase flows. This has led to increasing interest in alternative sensing technologies, such as Distributed Acoustic Sensing (DAS), to non-invasively assess flow characteristics and support real-time ICV management.

To overcome this limitation, DAS has been increasingly explored as a passive acoustic monitoring method for flow monitoring recently. DAS transforms standard optical fibers into dense arrays of acoustic sensors by detecting strain-rate signals along the fiber in response to flow-induced vibrations. Compared with traditional measurements like electronic-based sensing tools, the DAS system shows lots of advantages: it offers high spatial and temporal resolution, minimal invasiveness, and the ability to monitor well sections continuously [6,7,8].

Several recent studies have leveraged DAS to characterize multiphase flow behavior, identify flow regimes, and estimate fluid velocities. For instance, eddy tracking techniques utilize high-frequency signal fluctuations to estimate flow velocities [9], while Doppler-based methods infer changes in the speed of sound to determine flow velocities and composition [10]. More recently, machine learning models have been introduced to classify flow regimes or estimate flow parameters from DAS signals by extracting complex spectral or statistical features [11,12,13].

Despite the promise shown in prior studies, most DAS-based production monitoring approaches are limited in their applicability to intelligent completion systems. Many established techniques rely on conditions such as high flow rates or strong flow instabilities—necessary to resolve changes in the speed of sound (SoS) or to track eddies for velocity estimation. However, such phenomena are not consistently present across operating conditions, particularly in low-rate or stabilized production environments. Additionally, while vibration-intensity-based DAS methods—similar to noise logging—have been used for production allocation [14,15,16], these techniques often suffer from uncertainty due to their sensitivity to perforation geometry and completion-specific noise characteristics, which can vary widely and reduce result robustness [17]. These limitations point to a critical need for more physically grounded and generalizable DAS interpretation methods in complex downhole environments. In this context, Inflow Control Valves (ICVs) present a unique and underutilized opportunity. As engineered flow restrictions, ICVs introduce well-defined changes in flow geometry that consistently induce energy dissipation and acoustic emissions. Unlike unconstrained perforations or natural fracture entry points, ICVs provide a more controllable and repeatable relationship between flow behavior and acoustic signature, potentially enabling more accurate flow modeling and interpretation from DAS data.

Building on this opportunity, the present study investigates the Distributed Acoustic Sensing response in a horizontal well equipped with ICVs under oil–water two-phase flow. The novelty of this work lies in the integration of three components: an ICV-centered sensing configuration, DAS spectral feature extraction, and a physics-informed power-law model. First, the ICV is used as a controlled flow restriction that generates repeatable pressure loss and flow-induced acoustic energy, providing a more physically constrained sensing target than uncontrolled inflow or perforation noise. Second, DAS frequency band amplitudes (FBAs) are extracted from time–frequency spectrograms to quantify the acoustic response associated with different flow conditions. Third, instead of relying only on black-box machine learning models, we develop an interpretable power-law formulation that links DAS FBAs to pressure drop and flow rate. The fitted exponents are shown to be consistent with Bernoulli-type restriction-flow scaling, suggesting that DAS spectral amplitudes reflect flow-induced energy dissipation near the ICV. By analyzing controlled flow loop experiments, we demonstrate how DAS-derived frequency band amplitudes can be used to estimate pressure drop and flow rate with strong physical interpretability, while a $C_{v}$-based formulation is explored for water cut analysis, which remains challenging and is identified as a direction for future work. This study aims to bridge the gap between DAS technologies and practical production monitoring in intelligent well configurations. 

## 2. Experiment Setup

### 2.1. Experimental Facility and Setup

**Figure 1:**
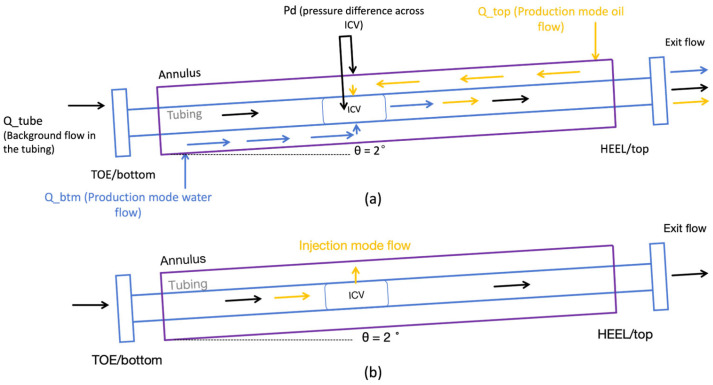
Schematic illustration of the experimental flow loop. (**a**) Production mode, (**b**) injection mode.

Two-phase (oil/water) flow loop experiments with the intelligent completion were conducted at the SINTEF facilities in Trondheim, Norway, from October 09 through 23 of 2023. The fluid properties of water used in testing were as follows: plain tap water at ambient temperature, with a reference density of 999 kg/m^3^ and a viscosity of 1 cP. The oil used was Exxsol D60, a de-aromatized hydrocarbon, with a typical density of 792 kg/m^3^ and variations within a temperature range of approximately ±5 kg/m^3^. The test section was designed to enable effective DAS signal capture during flow through the ICVs. It featured a concentric assembly of an inner tubing and an outer casing pipe, with the ICVs installed along the inner tubing. The outer casing had an outer diameter (OD) of 273 mm (10.75 in) and an inner diameter (ID) of 267 mm (10.5 in), while the inner tubing had an OD of 88.9 mm (3.5 in) and an ID of 77.9 mm (3.07 in). The full test section measured 24 m in length, with the tubing extending an additional meter beyond each end of the casing. The section was inclined at 2 degrees, resulting in a height difference of approximately 0.84 m from toe to heel. The outer casing was constructed from four 6 m segments, which were slid over the fiber- and sensor-equipped inner tubing during assembly. Pressure testing of the completed setup confirmed its integrity under experimental conditions. Continuous measurements of DAS, accelerometers, and temperature/pressure gauges were recorded at several locations along the flow loop for all tests. Although the tests were conducted generally at a controlled ambient temperature, small variations were observed. Most tests were performed at a system pressure of 5 bar. A cartoon sketch of the central test section, sensing device location, and flow direction for injection and production mode tests is provided in Figure 1. This study focuses exclusively on the production-mode experiments. The injection-phase experiments and additional experimental details are beyond the scope of this paper and are documented in the corresponding Halliburton’s report [18].

### 2.2. DAS Sensors and Measurement Setup

The flow loop was equipped with various sensors to acquire routine and continuous measurements of the apparatus and flow-generated signals. Pressure measurements were recorded at the inlet and outlet of the tubing, as well as the middle of the annulus, which also gave insights about the differential pressure between the tubing and the annulus. Temperature was monitored at the inlet and outlet of the tubing, as well as at the top and bottom of the annulus, using thermal gauges. Total flow rates for all fluid phases entering the tube were measured with a Coriolis meter, and the flow rate of water or oil entering the lower port on the annulus and oil and/or gas entering the upper port were also recorded. Typical measurement uncertainties included 0.09 °C for temperature and 0.1 bar for absolute pressure measurements, while the liquid phase flow rates had an uncertainty of 0.1%.

The design of fiber-optic sensor configuration along the flow loop was influenced by several factors, with a primary focus on comparing the experimental data to field cases that employed similar fiber installation configurations. The configuration also emphasized capturing the signals generated by the orifices of the flow control valves. In typical DAS field acquisitions for intelligent completions, a single-mode fiber runs along the length of the tubing. In this experiment, the setup included two 900 µm polyester tight-buffered (TB) ordinary single-mode fiber cables, running longitudinally along the flow loop tubing pipe. These linear fiber sections were complemented by helically wrapped sections near the flow ports to capture additional dynamic strain signals. The DAS interrogator used in these experiments records phase-difference measurements (for example, the differential phase shift between consecutive gauge lengths), meaning the acquired signals represent strain rate rather than absolute strain.

**Figure 2:**
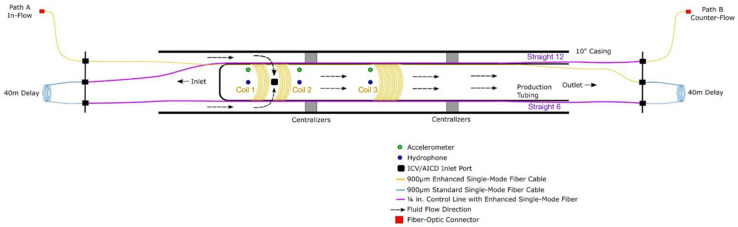
Schematic illustration of the fiber circuit installed for sampling with DAS (B. Schaeffer, 2024 [18]).

The installation of the fiber was carried out with caution to ensure optimal coupling to both the test flow device sections and the tubing pipe, as shown in Figure 2. For the DAS acquisition parameters, the system utilized a temporal sampling rate of 25 kHz, spatial sampling of 1.02 m, with a gauge length of 2.5 m.

## 3. Data Processing and Results

### 3.1. Raw DAS Data

**Figure 3:**
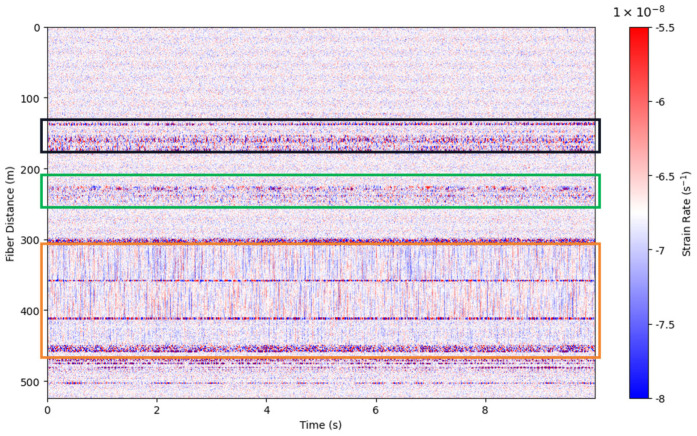
DAS strain-rate data over a 10 s window, highlighting cable sections: black (Straight 12), green (Straight 6), and orange (3 coils: Coil 3 comes first, followed by Coil 2 and Coil 1).

The production experiment was conducted over 7 days, during which all five fiber-optic cables were interrogated simultaneously, with the cables connected in sequence. Additionally, flow loop sensor data—including pressure, flow rates, and temperature—was logged at a time resolution of 0.5 s. A 10 s segment of raw DAS data from all cables is presented in Figure 3. The fiber segmentation is defined as follows:

1. Straight 12 cable: Positioned along the tube at the 12 o’clock direction, covering fiber distances from 154 m to 178 m, with flow direction from 178 m to 154 m;
2. Straight 6 cable: Positioned along the tube at the 6 o’clock direction, covering fiber distances from 225 m to 249 m, with flow direction from 225 m to 249 m;
3. Coil 3 cable: Helically wrapped near the heel of the tube, spanning fiber distances from 310 m to 358 m, with flow direction from 358 m to 310 m;
4. Coil 2 cable: Helically wrapped at the heel side of the ICV, covering fiber distances from 362 m to 411 m, with flow direction from 411 m to 362 m;
5. Coil 1 cable: Helically wrapped at the toe side of the ICV, spanning fiber distances from 416 m to 449 m, with flow direction from 449 m to 416 m.

Due to the high spatial and temporal resolution of DAS data, the raw data volume is extremely large up to 88 GB per hour of recording. Such massive datasets pose significant challenges for direct interpretation and necessitate an efficient dimensionality reduction strategy. To enhance interpretability and reveal flow-regime-dependent features, frequency-domain spectral analysis was employed.

### 3.2. Spectrum Analysis

To convert raw 2D time–distance DAS data into a format that captures both temporal and spectral dynamics, a Short-Time Fourier Transform (STFT) approach was used. STFT involves dividing the time series into non-overlapping windows—in this case, 10 s segments—and computing the Fast Fourier Transform (FFT) for each segment. This process introduces a third dimension (frequency), transforming the raw 2D DAS data (distance × time) into a 3D tensor, distance × time × frequency, where each value corresponds to the amplitude of the signal at a specific channel, time, and frequency.

Unlike broadband PSD calculations that square FFT coefficients and integrate energy over frequency intervals, this study works directly with the complex FFT coefficients returned by the STFT. The frequency bins are defined by the discrete Fourier frequencies without squaring or bandwidth-normalization, so the resulting spectra reflect relative amplitude distributions rather than absolute power spectral density. This approach is chosen to preserve the shape of the DAS frequency response, which is used as a diagnostic feature rather than an energy-normalized PSD [19].

To focus the analysis on specific cable segments, the 3D FFT data were subset according to the five fiber sections described above. Within each section, the deployment geometry and sensor placement are consistent, and the DAS signal is expected to exhibit high coherence along the spatial channels. Therefore, the spectral amplitudes across channels within each section were averaged along the distance dimension. This reduces the 3D tensor to a 2D spectrogram (frequency × time) for each fiber section. These spectrograms provide time-resolved insight into the acoustic energy distribution across frequency bands and are used to monitor the stability of the DAS response during each test.

Although the spectrograms reduce the data by one dimension, the time axis still contains a very large number of samples due to the long duration of the experiment (about 10 h a day over 7 days). To further reduce data size and focus on representative flow conditions, we transitioned from continuous time to test-based analysis. During the 7-day-long production experiment, 118 tests were designed to cover a wide range of flow rates, water cut (WC), and ICV valve position. Here, each test has a period of 5 to 10 min during which stable flow conditions were maintained, and flow/differential pressure measurements were obtained. Exxsol D60 and freshwater were used as test fluids. The testing matrix included 32 single-phase oil, 8 single-phase water, and 78 two-phase oil–water tests. Throughout the experiments, the WC ranged from 0 to 100%, and the total liquid rate ranged from 0.65 to 20.07 m^3^/hr. To achieve the desired viscosities, the loop temperature was adjusted accordingly.

Given that the flow is stable during each test period, each spectrogram was averaged over time within each test, resulting in a 1D spectrum (frequency) for each fiber section and test. To ensure the quality of the averaging process and exclude unstable effects at the beginning or end of a test period, a time window starting 60 s after test initiation and ending 60 s before test termination was selected for averaging.

**Figure 4:**
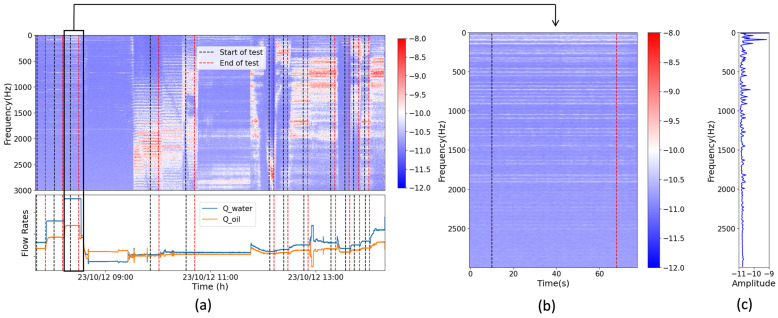
DAS spectral data processing workflow. (**a**) displays the spectrogram of DAS data (Straight 12) along with flow rates recorded during the fourth day of experiments. The black and red dashed lines indicate the start and end times of formal tests. (**b**) presents an example of a spectrogram from a single formal test. (**c**) shows the averaged spectrum of the formal test, which is used to represent the spectrogram in subsequent analysis.

While one could theoretically compute the FFT directly over each test window, the spectrogram offers an important visual verification of flow regime stability. The consistency of spectral features across time confirms that the averaged spectrum is representative of steady-state flow conditions. This step is crucial before proceeding to statistical modeling. The overall data processing workflow using the fourth day’s data from the Straight 12 fiber as an example is summarized in Figure 4. Specifically, the spectrogram is divided into several test windows as illustrated in Figure 4a. By zooming into each window, we observe that the spectrogram within an individual test period remains stable, as shown in Figure 4b. Finally, these stable spectrograms are averaged over time to yield a mean stable spectrum.

**Figure 5:**
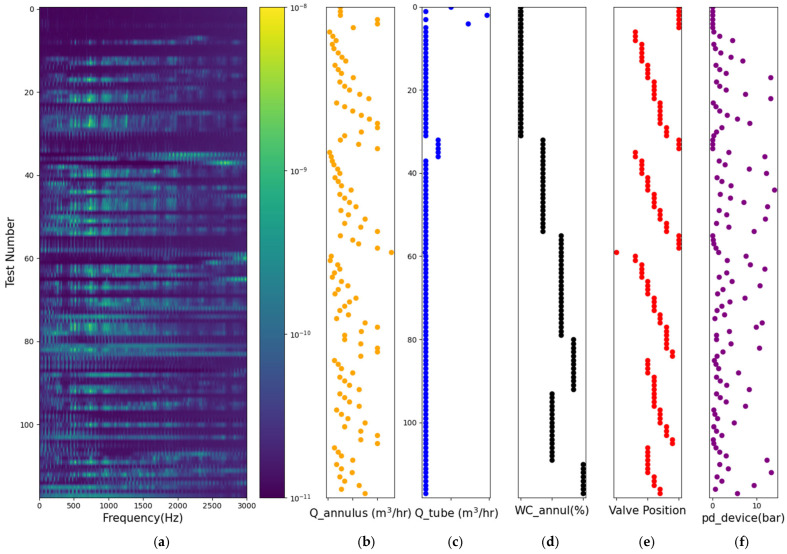
(**a**) A combined view of FFT spectra of Straight 12 cable, (**b**) mixture flow rate in annulus, (**c**) mixture flow rate in tube, (**d**) water cut (WC) in annulus, (**e**) valve position of ICV that indicates the valve opening, and (**f**) pressure drop across ICV.

Following this data preparation, we computed the mean values of key flow parameters—such as pressure drop across the ICV, annular and tubing flow rates, valve opening, and water cut—for each test, during the same time period as the averaged DAS data. These averaged flow parameters were then compared with the corresponding DAS-derived spectral features. The results are summarized in Figure 5, which provides a consolidated view of how the DAS signal responds under different flow conditions across all tests. Notably, the figure shows that as liquid flow rate increases, both the pressure drop across the ICV and the DAS spectral amplitude exhibit a monotonic increase, illustrating the strong coupling between DAS response and flow dynamics.

### 3.3. Flow Parameter Estimation

In order to better estimate the oil–water flow profile using DAS measurements, this section explores the influencing correlation between flow parameters (rate, water cut) and the DAS signals.

#### 3.3.1. Pearson Correlation Analysis

The Pearson correlation coefficient method was first applied to evaluate the relationship between DAS spectral amplitudes and key flow parameters, including pressure difference across the ICV (Pd), injected flow rate ($Q_{annulus}$), injected water cut (WC), valve position (opening), and background tubing flow rate ($Q_{tube}$). This analysis was used to identify which flow parameters and frequency bands were most strongly related to the DAS response before applying multivariate and physics-informed modeling.

**Figure 6:**
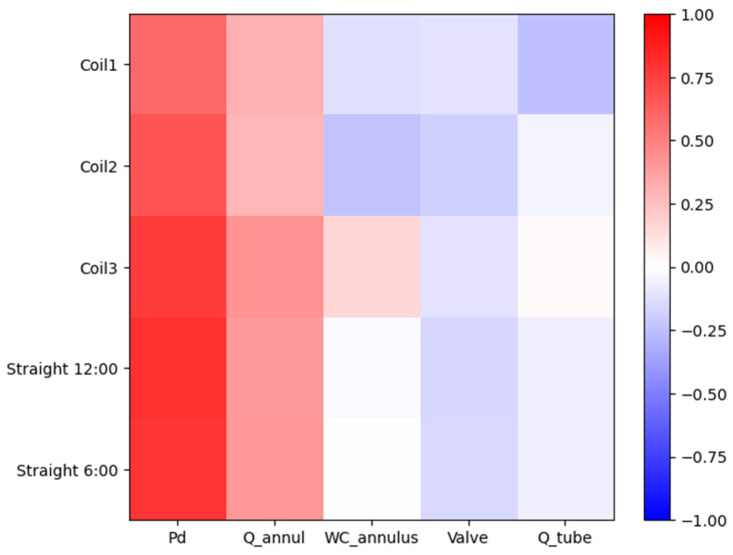
Pearson correlation coefficient between DAS average spectral amplitude between 0 and 3000 Hz and flow parameters.

Figure 6 shows the correlation between the averaged DAS spectral amplitude from 0 to 3000 Hz and the selected flow parameters for all five fiber sections. The results indicate that $P_{d}$ has the strongest correlation with the DAS signal, especially for the two straight fiber sections. Flow rate shows a moderate correlation, while water cut, valve opening, and background tubing flow rate show relatively weak correlations. These results suggest that the DAS response is most sensitive to pressure-loss-related flow energy near the ICV.

**Figure 7:**
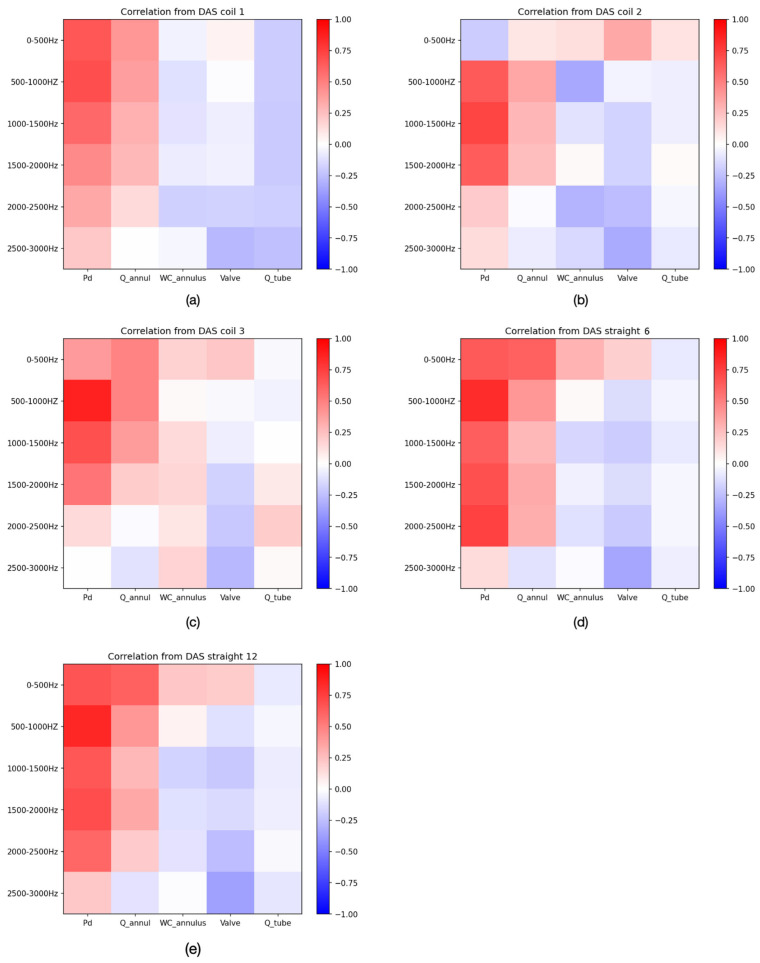
Pearson correlation coefficient between DAS spectral frequency band amplitude and flow parameters of different fiber cables: (**a**) Coil 1, (**b**) Coil 2, (**c**) Coil 3, (**d**) Straight 6, and (**e**) Straight 12.

To further examine frequency-dependent behavior, the DAS spectra were divided into six 500 Hz wide frequency bands from 0 to 3000 Hz, and the correlation between each frequency band amplitude (FBA) and the flow parameters was calculated for each fiber section. As shown in Figure 7, the 500–2000 Hz bands generally show stronger correlations with $P_{d}$ and Q, particularly in the straight fiber sections. This indicates that the acoustic energy associated with flow through the ICV is mainly captured within this frequency range. Therefore, these FBAs were selected as key input features for the subsequent analyses.

#### 3.3.2. Muti-Variance Results

While the previous Pearson correlation analysis used frequency band amplitudes (FBAs) to evaluate the linear relationship between individual flow parameters and DAS signals, it was limited to analyzing pairwise dependencies. That is, although it showed that pressure difference (Pd) is strongly correlated with specific frequency bands—especially in the straight and Coil 3 sections—it could not capture combined or nonlinear relationships among multiple flow parameters and spectral features.

To address these limitations, we applied a machine learning-based multivariate analysis (MVA) approach to evaluate how combinations of multiple FBAs and operational parameters influence key flow outcomes. Multivariate analysis (MVA) is a feature selection and model evaluation workflow designed to identify the most informative and correlated variables within complex, high-dimensional datasets. Originally demonstrated by Ning et al. (2023) [20], the MVA framework iteratively evaluates model performance using machine learning, typically with Random Forests and cross-validation strategies, to rank combinations of features based on predictive accuracy. The workflow of MVA begins by training a machine learning model on each individual feature and assessing its performance using K-fold cross-validation with the R^2^ score (coefficient of determination) which is the proportion of the variation in the dependent variable that is predictable from the independent variable. It then evaluates all possible combinations of feature pairs, triplets, and higher-order subsets, selecting those that maximize predictive performance. This staged approach ensures that the final selected features offer both high relevance and low redundancy. By systematically ranking features and feature combinations, MVA provides a data-driven foundation for interpreting DAS signal relationships and guiding model development. In this study, it helped assess the relative importance of valve setting and DAS spectral features in estimating flow parameters and supported the identification of promising input variables.

In this study, the MVA method was applied to explore the relationship between DAS-derived features and flow parameters like pressure drop and flow rate, with the goal of identifying which variables or combinations of variables are most informative for flow parameter estimation. The spectral features used here were the same as those introduced in the previous correlation analysis: for each of the five fiber sections, spectral amplitudes were extracted across six 500 Hz wide frequency bands spanning 0–3000 Hz.

**Figure 8:**
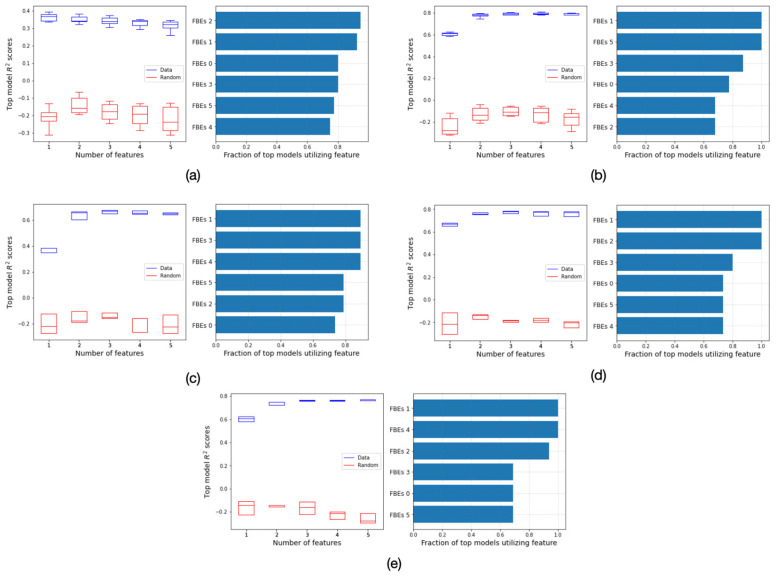
Multivariate results for 100 iterations of the MVA workflow on estimating pressure difference from five cable sections: (**a**) Coil 1, (**b**) Coil 2, (**c**) Coil 3, (**d**) Straight 12 and (**e**) Straight 6. The left panel under each group shows box-plots of the R^2^ scores at each number of features (original data in blue and shuffled data in red) along with the optimal number of features listed on right. The right panel under each section shows the fraction of the top models that included each feature, indicating the importance of that feature.

The MVA results of pressure difference (Pd) and injected flow rate (Q) are depicted in Figure 8, and a summary of the MVA results for all flow parameters is provided in Table 1. Consistent with the Pearson correlation analysis, Pd exhibits a high R^2^ score with the two straight and Coil 3 cable sections. In addition, MVA also exhibits the importance of different FBA features for Pd estimation. As Figure 8 shows, FBA1 (500–1000 Hz) shows much higher importance for all five cable sections, which suggests the acoustic signal introduced by the pressure drop across the ICV is more likely in the 500 to 1000 Hz frequency band. For flow rate (Q) and water cut (WC) predictions, the MVA revealed an additional insight: when valve opening was included as an input, the DAS FBA demonstrated strong predictive capability for the flow rate, achieving an R^2^ score of approximately 0.8. This finding suggests a new way to estimate flow rate using DAS signals within the valve opening information. However, for water cut prediction, the R^2^ scores remain low across all five cable sections, indicating poor predictive performance. Figure 6 also illustrates these limitations. Notably, when the water cut varied during the tests, the spectral amplitude recorded by DAS did not exhibit a clear trend corresponding to the changes in water cut. This suggests that, compared to flow rate and valve opening, water cut has a weaker influence on the DAS signal.

**Figure 9:**
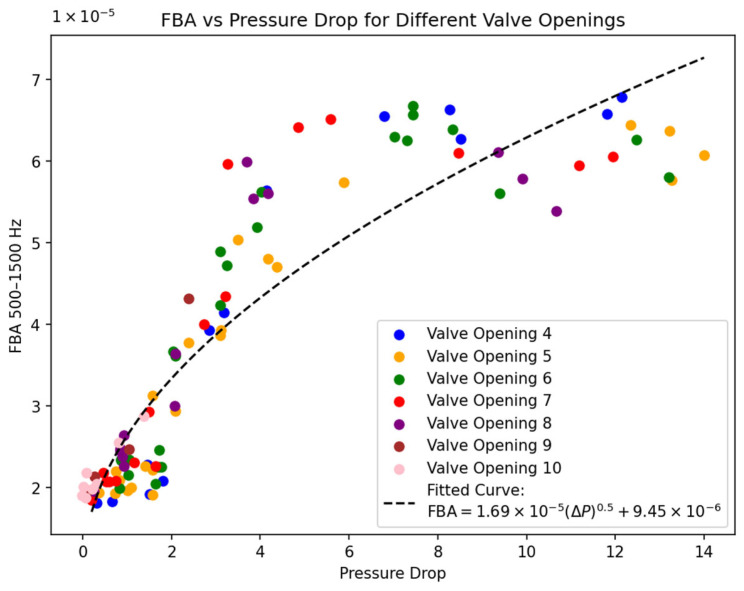
Computed DAS frequency band amplitude (500–1500 Hz) compared with pressure difference for different ICV valve positions from Straight 12 fiber cable section. The black dashed line shows the fitted curve using all data points.

**Figure 10:**
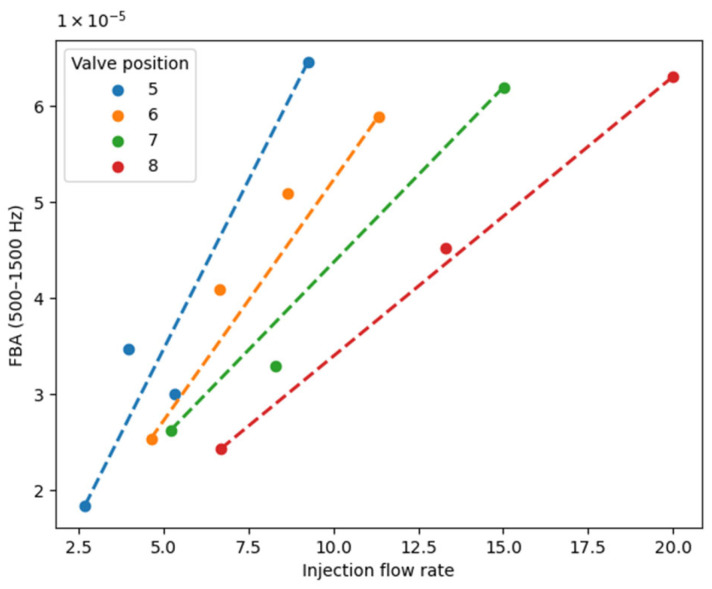
Computed DAS spectral amplitude (500–1500 Hz) with respect to flow rates for four different ICV positions and same water cut 35% from Straight 12 fiber cable section, with trend lines annotated.

From correlation analysis, it is found that the DAS spectral amplitudes are highly correlated with Pd, Q, and valve open information. However, the MVA machine learning workflow functions as a black box and does not clearly reveal the relationships between the flow parameters and FBAs. And it is also hard to apply to other datasets due to the limited generalization capability of the machine learning models. So, it is important to further analyze the quantified relationships. To better understand the relationships, we plot Pd and Q with FBAs in a range of 500 to 1500 Hz from the Straight 12 cable section, in which frequency bands show a higher importance in MVA. The corresponding cross plots are presented in Figure 9 and Figure 10. As shown in Figure 9, for 118 test points, a general positive nonlinear relationship between the FBA from 500 to 1500 Hz and Pd was observed. However, the scatter becomes more noticeable in very low and very high pressure drop ranges. At low $P_{d}$, the injected flow rate and flow-induced acoustic energy are relatively small, so the DAS response is closer to the background noise level, resulting in a lower signal-to-noise ratio and less stable FBA measurements. At high $P_{d}$, stronger restriction-induced turbulence, local flow instability, and possible flow-regime transitions may introduce additional variability in the DAS response. Then, to better understand the impact of ICV valve opening area, four groups of tests with different valve position settings are selected as shown in Figure 10. A near-linear relationship is observed between the DAS signal amplitude and injected flow rate ($Q_{annulus}$), where the slope of this relationship becomes steeper as the valve opening decreases and the flow area is further restricted. These relationships demonstrate promising potential for the measurement of pressure difference and injected flow rate.

#### 3.3.3. Physics-Based Predictive Models

In this section, we will develop an interpretable and physically grounded model for estimating pressure drop and flow rate from DAS spectral features.

DAS provides indirect measurements of flow behavior through its sensitivity to flow-induced vibrations. In the Experimental section, we observed that the DAS spectral amplitude increases monotonically with both the pressure drop and flow rate, suggesting that the DAS signal effectively captures the underlying changes in flow energy and momentum. This observation motivates a regression formulation in which pressure drop and flow rate are modeled as power-law functions of DAS frequency band amplitudes (FBAs) and the valve opening area (A). The goal is to develop mathematical models that retain interpretability and reflect the underlying physical dependencies. The two primary models are expressed in Equations (1) and (2):

(1) $$Pd=\sum_{i}\alpha_{i}FBA_{i}^{n_{1}},$$

where Pd denotes the pressure difference across the ICV, and $FBA_{i}$ represents the amplitude of the i-th DAS frequency band. The coefficient α varies for each frequency band, while n_1_ defines the power of FBAs.

(2) $$Q=\sum_{i}\beta_{i}FBA_{i}^{n_{2}}A^{n_{3}},$$

where Q denotes the flow rate across the ICV, and A represents the opening area of the ICV. The coefficient β varies for each test, while n_2_ defines the power of the FBA and n_3_ defines the power of area.

Based on MVA, it was found that the pressure drop (Pd) could be reasonably well estimated using the DAS-derived spectral features (FBAs) alone, without requiring information about the valve opening area (A). This suggests that the acoustic energy associated with pressure losses across the ICV is sufficiently captured by the spectral response alone. In contrast, flow rate (Q) estimation is more complex: satisfactory prediction accuracy could only be achieved when both FBAs and the valve opening area were included as input features. Consequently, in the mathematical models, the area term is excluded from the Pd regression but incorporated into the Q formulation to reflect these differing sensitivities and ensure consistency with both data-driven insights and physical principles.

Unlike conventional polynomial regression, which typically includes combinations of multiple power terms of each variable, this formulation imposes a single power exponent per variable to preserve physical interpretability. This avoids overfitting and prevents conflation of effects due to multiple overlapping powers, which can obscure insights about which features dominate system behavior. Moreover, from a physical standpoint, the relationship between pressure drop and flow rate is expected to follow a single power-law dependency rather than a combination of multiple power terms. Therefore, the mathematical models not only provide better interpretability but also align more closely with the fundamental physics governing the oil–water two-phase flow system.

This constrained power-law form allows for direct comparison of models across different exponent combinations. The model ensures that only exponent terms are used for each variable. By evaluating the R^2^ scores of models using different exponent values, we can identify the exponent that best captures the underlying physics.

To construct physical-based mathematical models, we focused on estimating pressure drop (Pd) and total flow rate (Q), which show highest correlation with FBAs, using DAS-derived frequency band amplitudes, and the same data ranges applied in the Pearson correlation and MVA analyses. The same approaches were applied to all fiber sections.

For the pressure drop predictive model, we tested a series of exponent values (n_1_) applied to the frequency band amplitude (FBA). A unique coefficient $\alpha_{i}$ is associated with each n_1_, representing its best fit between pressure drop and the FBA. Similarly, for the flow rate predictive model, we constructed features in the form: $FBA^{n_{2}}\cdot A^{n_{3}}$. Each unique combination of n_2_ and n_3_ produces a different set of transformed features, which in turn results in a distinct coefficient $\beta_{i}$ that best characterizes the relationship between the features and the flow rate. To systematically search for the best-fitting model, linear regression was then applied to each model, and the performance was evaluated using the R^2^ score. By comparing R^2^ scores across all tested exponent combinations, the optimal model was identified as the one with the highest R^2^ score (i.e., the lowest prediction error).

**Figure 11:**
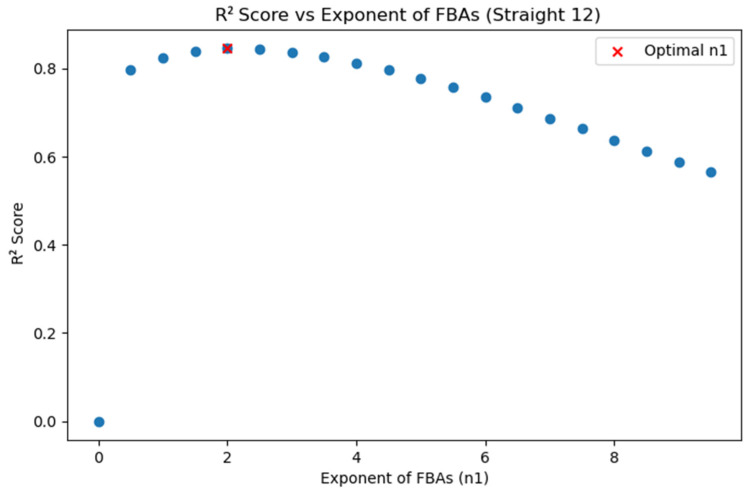
R^2^ score of Pd model with different exponents of FBAs for fiber section Straight 12.

The optimal model for pressure drop prediction was obtained when $n_{1}=2$, aligning well with the Bernoulli equation where pressure loss is proportional to the square of flow velocity. Figure 11 illustrates how the R^2^ score changes with $n_{1}$ for the Straight 12 fiber section, showing a clear peak at $n_{1}=2$. This component is also consistent with the nonlinear trend observed in Figure 9, where FBA scales approximately with the square root of pressure drop, implying a quadratic relationship between pressure drop and the FBA.

Table 2 summarizes the determined optimal coefficients $\alpha_{i}$ across different frequency bands and fiber sections. All fiber sections achieved the best performance when $n_{1}=2$. With this observation, the pressure drop estimation model can be expressed as Equation (3):

(3) $$Pd=\sum_{i}\alpha_{i}FBA_{i}^{2}.$$

Notably, frequency bands between 500 and 2000 Hz consistently contributed the most to pressure drop estimation for all fiber sections, indicating that a majority of the acoustic energy generated by flow through the ICV falls within this range. Among all fiber sections, the model from the Straight 12 section produced the highest R^2^ score, highlighting its superior sensitivity to the differential pressure.

**Figure 12:**
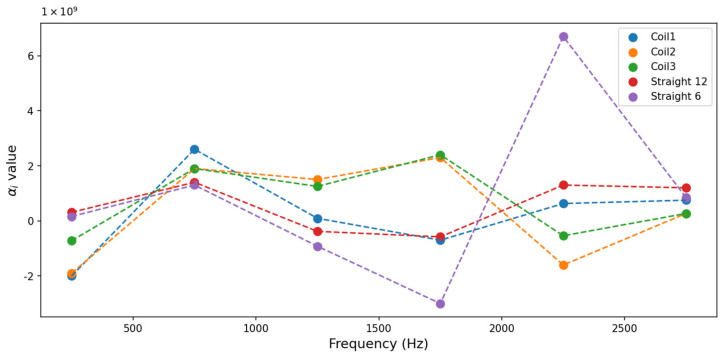
Plot of coefficients (used to calculate Pd, per Equation (3)) for different frequency bands and fiber sections. The colored dashed lines represent the trends of α values for different fiber sections, including Coil 1, Coil 2, Coil 3, Straight 12, and Straight 6.

Figure 12 further explores how the regression coefficients vary with frequency and fiber placement. Coils 2 and 3 exhibit consistent trends, suggesting similar acoustic coupling conditions despite their helical geometry. Coil 1 behaves differently from Coils 2 and 3 because it was positioned upstream of the flow intake point. The two straight sections also display a different trend compared to Coils 2 and 3, likely due to their distinct deployment method and coupling configuration. Such differences in placement can influence how acoustic energy is transmitted and captured along the fiber.

**Figure 13:**
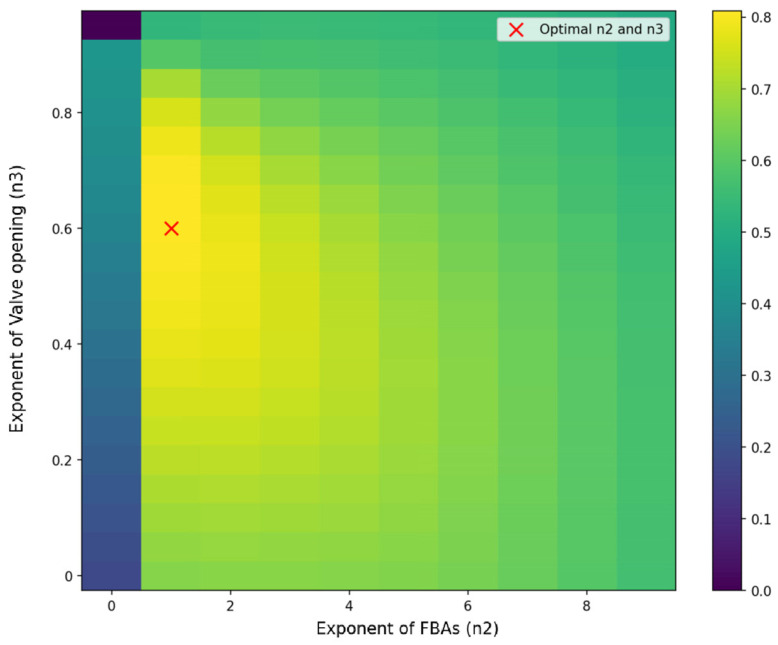
$R^{2}$ scores for different exponents of FBA and valve opening for the Straight 12 cable. The color bar represents the $R^{2}$ scores.

For flow rate estimation, the best $R^{2}$ score of the power-law models yielded the optimal coefficients, $n_{2}$ and $n_{3}$. Figure 13 shows the optimized $R^{2}$ scores with changing $n_{2}$ and $n_{3}$ values of the Straight 12 cable, and the highest $R^{2}$ is achieved when $n_{2}$ equals one and $n_{3}$ is around 0.6; this optimal exponent is consistent with all cable sections. As a result, the flow rate across the ICV measurement model can be expressed in (4):

(4) $$Q=\sum_{i}\beta_{i}FBA_{i}A^{0.6},$$

**Figure 14:**
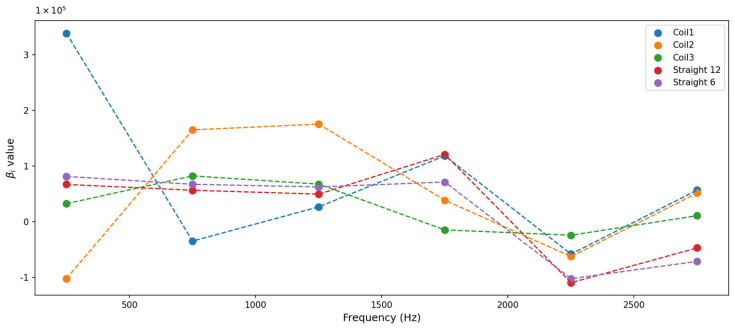
Plot of β coefficients (used to calculate Pd, per Equation (4)) for different frequency bands and fiber sections. The colored dashed lines represent the trends of coefficient values for different fiber sections, including Coil 1, Coil 2, Coil 3, Straight 12, and Straight 6.

Table 3 shows all β coefficients for all five cable sections with optimized $n_{2}$ and $n_{3}$. Figure 14 presents the variation in β across different frequency bands and DAS fiber sections. The two straight fiber sections exhibit consistent β patterns, confirming the stability of the DAS response in straightly deployed configurations. In contrast, the coiled sections display distinct behaviors: Coils 2 and 3 follow similar trends but differ in amplitude, whereas Coil 1 deviates more notably due to its upstream position relative to the intake point. This positional influence, combined with the complex coupling introduced by the helical wrapping, explains the observed variation among coils. The overall similarity between the α and β trends further validates the robustness of the power-law regression framework and underscores the role of fiber geometry in shaping the DAS signal response.

#### 3.3.4. Model Evaluation

**Figure 15:**
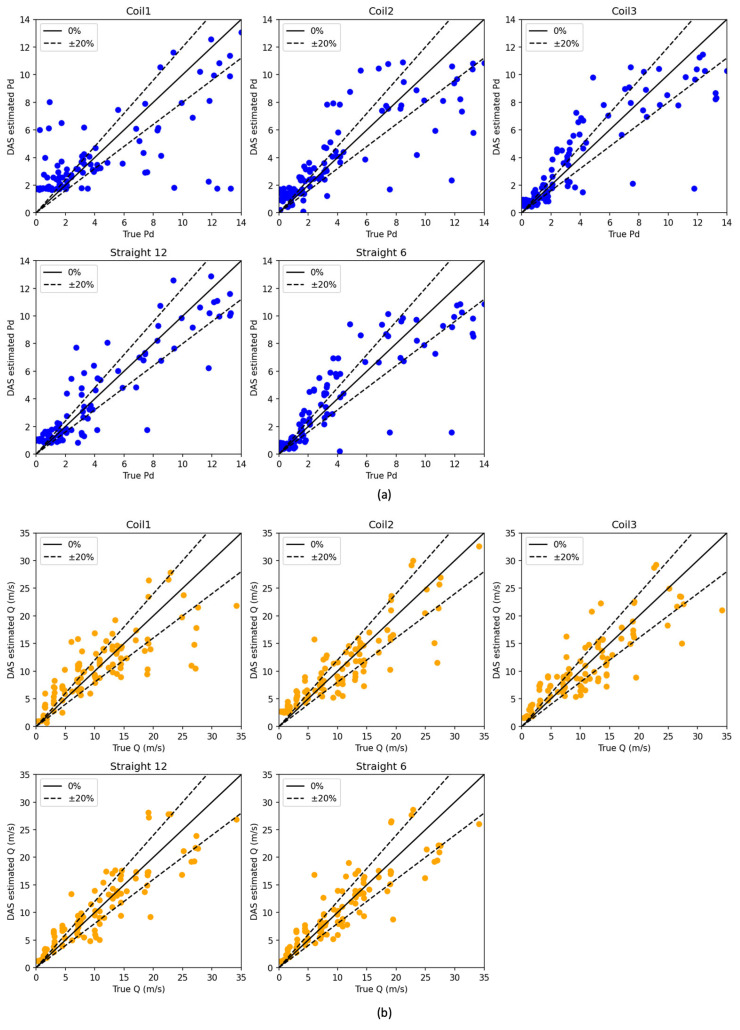
Cross plots comparing model predictions with measurements for all cables. (**a**) Blue point show Pressure difference, (**b**) Orange points show flow rate across ICV. *X*-axis is the true Pd and Q values from experiment sensor measurement, *Y*-axis is Pd and Q values from DAS-based predictive models.

To further evaluate the performance of the proposed mathematical models, cross plots were generated for all tests from five fiber cables. A comparison of estimated versus measured median values for pressure difference and flow rate is shown in Figure 15. Across the 118 tests with a wide range of flow conditions, the model showed generally good agreement with the reference sensor, with most estimates within a 20% error margin. These results suggest that DAS-based predictive models have promising potential for estimating both pressure drop and flow rate under varying flow conditions.

It is worth noting that the proposed physics-based mathematical model achieved an $R^{2}$ comparable to that of the machine-learning-based models (Table 1 and Table 2). This implies that the proposed models are capable of capturing the causal correlations between the DAS measurements and flow parameters, and that they hold potential for generalization for field deployment.

However, a slight systematic bias is observed at low values of pressure drop and flow rate. These discrepancies are likely associated with the lower signal-to-noise ratio (SNR) in the DAS data under low flow rate conditions, where the acoustic energy generated by the flow becomes comparable to background noise. Under such conditions, the sensitivity of spectral features to flow-induced dynamics is reduced, underscoring the importance of the signal-to-noise ratio (SNR) in DAS-based flow monitoring.

## 4. Discussion

### 4.1. Physical Insights into DAS Spectral Signal–Flow Parameters (Q and Pd) Correlations

In this section, we interpret the meanings of the fitted coefficients and exponents in the context of flow dynamics, energy dissipation, and acoustic response, and discuss how these insights enhance our understanding of DAS-based predictive models and their application in real-time flow monitoring.

A particularly noteworthy observation from the predictive models for pressure drop (Pd) and flow rate (Q), expressed in Equations (3) and (4), is the power-law relationship between the DAS frequency band amplitude (FBA) and the flow parameters. Specifically, the pressure difference is proportional to the square of the FBA, while the flow rate is proportional to its first order, as shown in Equation (5):

(5) $$d∼FBA^{2}\;and\;Q∼FBA,$$

This indicates that the pressure difference should be proportional to the square of the flow rate, as given in Equation (6):

(6) $$Pd∼Q^{2},$$

This relationship is consistent with Bernoulli’s equation, which relates pressure drop to changes in kinetic and potential energy, as expressed in Equation (7):

(7) $$P_{1}+\frac{1}{2}\rho v_{1}^{2}+\rho gh_{1}=P_{2}+\frac{1}{2}\rho v_{2}^{2}+\rho gh_{2}$$

Several studies [21,22,23,24] have derived equations predicting the pressure drop across restrictions such as ICVs, chokes, or nozzles as a function of in situ velocity from Bernoulli’s equation, showing that the pressure drop is proportional to the square of velocity:

(8) $$P\propto v^{2},$$

Given that $v=\frac{Q}{A}$, the pressure drop across the restriction is proportional to the square of volumetric flow rate:

(9) $$\Delta P\propto {\left(\frac{Q}{A}\right)}^{2}\propto \;Q^{2}\;,$$

This similarity suggests that the power-law models are not only empirically accurate but also grounded in well-established physical principles. From a fluid mechanics perspective, the pressure drop across the ICV represents energy loss caused by the restriction, as the fluid accelerates through the reduced flow area. The results indicate that the DAS spectral amplitude is proportional to the square root of this energy loss, meaning that higher kinetic energy losses result in stronger acoustic responses captured by the fiber.

The regression results are consistent with this relationship, reinforcing the interpretation that the DAS amplitude serves as a proxy for flow-induced energy dissipation. This agreement between empirical regression results and fundamental fluid dynamic theory supports the validity and generalizability of our approach.

Overall, these findings demonstrate that DAS-based spectral features can reliably capture physically meaningful flow behavior in oil–water systems, particularly in quantifying the relationship between flow rate and pressure loss across ICVs. This provides a foundation for extending DAS applications to real-time, physics-informed monitoring of multiphase production systems.

### 4.2. Physical Insights into DAS Spectral Signals’ Correlations with Water Cut and Valve Opening

To better understand the physical relationship among pressure drop, flow rate, valve opening, and water cut, we analyze the hydraulic behavior using independent laboratory measurements rather than the DAS-derived FBA. In this section, $P_{d}$ is the pressure drop measured by pressure sensors, Q is the experimentally measured flow rate, and valve opening and water cut are obtained from the experimental test conditions. Therefore, the following analysis is not a repeated DAS-based regression, but a hydraulic interpretation used to explain the optimized exponents obtained in the DAS-based power-law model. We begin with the classic model defining the pressure drop across a restriction for the liquid phase, given in Equation (10) [25].

(10) $$q_{L}=C_{D}A_{ch}\sqrt{\frac{2\Delta P}{\rho_{L}}},$$

where $q_{L}$ is the liquid flow rate, $C_{D}$ is the discharge coefficient, Ach is the choke flow area, ΔP is the pressure drop across the choke, and $\rho_{L}$ is the fluid density. This equation reflects the conversion of pressure energy into kinetic energy as fluid accelerates through restrictions.

Inspired by this principle, we adopt a simplified form that captures the quadratic relationship between pressure drop and flow rate in our system. Specifically, we introduce a comprehensive coefficient, $C_{v}$, that is applied to lump together all other parameters influencing the system.

(11) $$Pd=C_{v}Q^{2},$$

where $C_{v}$ encompasses the effects from multiple parameters, such as geometric factors including valve opening, the discharge coefficient, and fluid properties such as density.

**Figure 16:**
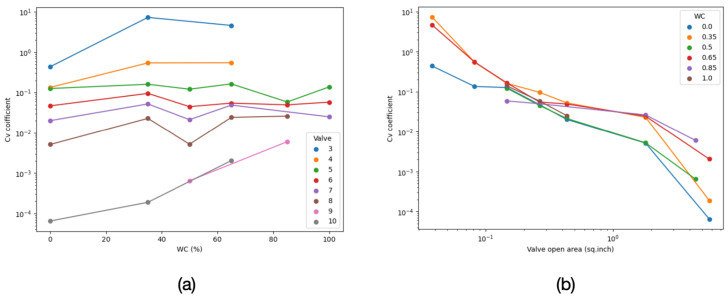
$C_{v}$ coefficients from experiment data vs. WC and open area. (**a**) Water cut, where different colored solid lines represent different valve openings. (**b**) valve open area. where different colored solid lines represent different water cuts.

In this study, $C_{v}$ can be represented as a function of water cut and ICV opening. This formulation enables a practical way to characterize flow behavior in terms of measurable quantities and to relate them to DAS spectral features. In this context, $C_{v}$ is not constant but varies with operational conditions; it can be expressed as a function of the ICV valve opening (A) and water cut (WC), i.e., $C_{v}=f\left(A,WC\right)$. To further investigate how $C_{v}$ varies with water cut and valve opening, we plot $C_{v}$ against these variables in Figure 16. The results indicate that $C_{v}$ decreases proportionally with increasing valve opening, regardless of water cut. This trend is expected, as for tests with the same injected flow rate and water cut, a larger valve opening reduces flow restriction, leading to lower energy dissipation and, consequently, a smaller pressure drop. However, the relationship between $C_{v}$ and water cut is more complex and nonlinear. Across the five tested water cut settings, $C_{v}$ initially increases from 0% to 35%, decreases to 50%, then increases at 65%, before finally decreasing as water cut approaches 100%. This behavior indicates that water cut may affect not only the mixture density but also the in situ Reynolds number, rheological properties, and flow pattern of the two-phase system, thereby contributing to the complex variation in pressure gradient with water cut.

**Figure 17:**
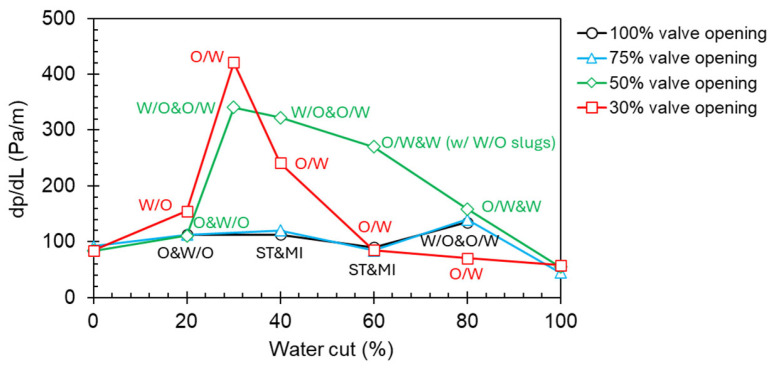
Pressure gradient in a pipe downstream of a choke as a function of water cut at various choke openings (Zhou et al. 2024 [26]).

This nonlinear trend closely resembles the variation in oil–water two-phase flow pressure drop with water cut in a pipe downstream of a choke, as reported by [26] and illustrated in Figure 17. Specifically, high pressure drops are observed around 30% and 80% water cut, while a lower pressure drop occurs at intermediate water cuts. Although the tested facility systems and operating conditions are not identical, the results could offer valuable insights relative to the current study.

To better explain how the DAS signal responds to the valve opening, we express $C_{v}$ in the form of:

(12) $$C_{v}=f_{1}\left(WC\right)\cdot f_{2}\left(A\right),$$

where $f_{1}\left(WC\right)$ captures the nonlinear effect of water cut on flow behavior, and $f_{2}\left(A\right)$ represents the influence of valve opening area.

Figure 16b previously shows the decreasing trend of coefficient $C_{v}$ with valve opening area. In this study, $f_{2}$ is presented as:

(13) $$f_{2}\left(A\right)=A^{n_{5}},$$

This gives the expression of $C_{v}$ as:

(14) $$C_{v}=f_{1}\left(WC\right)\cdot A^{n_{5}},$$

The same regression analysis used for pressure and flow rate estimation is applied to determine the optimal value of n_5_. Water cut and valve opening are treated as input features, while the $C_{v}$ coefficients back calculated from measured flow parameters serve the target values. We find that n_5_ is around −1.2, as shown in Table 4, which lists the optimal n_5_ across multiple water cut conditions.

With this, the pressure drop using average n5 values from Table 4 can be expressed as:

(15) $$P_{d}=f_{1}\left(WC\right)\cdot A^{-1.26}\cdot Q^{2},$$

This finding aligns well with the DAS-based models, given in Equations (4) and (6). This is explained by Equation (16) below. When substituting Equation (6) into (15), A cancels out, leading to $Pd\propto {FBA}^{2}$, which is consistent with the observations in Equation (4).

(16) $$\begin{array}{ll} Pd & =C_{v}Q^{2}=f_{1}\left(WC\right)\cdot A^{-1.26}\cdot Q^{2}\\ & =f_{1}\left(wc\right)\cdot A^{-1.26}\cdot {\left(FBA\cdot A^{0.6}\right)}^{2}\\ & \approx f_{1}\left(wc\right)\cdot FBA^{2}\propto FBA^{2}. \end{array}$$

This consistency of the exponents across both the physical and data-driven models suggests that valve opening has a sublinear influence on flow rate due to the nonlinear hydraulic behavior of flow through the ICV restriction. It also reinforces the physical interpretability of our DAS-based models: the fiber-optic signal amplitude reflects not only flow intensity but also the geometric constraints imposed by valve settings. Together, these results highlight the value of combining DAS spectral features with physically informed regression structures to gain insight into multiphase flow behavior and ICV dynamics under various operating conditions.

### 4.3. Discussion, Limitations, and Future Application Considerations

While the power-law models framework developed in this study demonstrates strong predictive power under controlled laboratory conditions, its direct application to field deployments requires careful consideration. The coefficients (e.g., α and β) derived from lab data are influenced by specific experimental hardware, flow loop geometry, and fluid properties—and thus may not be directly transferable to actual well environments where hardware configurations, reservoir properties, and operating conditions vary significantly.

In real-world intelligent wells equipped with multiple Inflow Control Valves (ICVs), one major challenge is that the flow rate through each ICV is unknown. Unlike in the lab, where individual test segments provide isolated flow measurements, field operations typically only offer surface-level measurements of total production rate. However, independent calibration under field conditions is not completely infeasible. When operating a single ICV at a time, zonal flow rates can be measured at the surface or downhole using inflow logging tools (ILTs), enabling calibration if operationally permitted. While calibration is possible, it may be operationally challenging and dependent on well-specific conditions.

Results from this study provide an encouraging insight: the similarity observed between regression coefficients derived from the two straight fiber sections (6 o’clock and 12 o’clock) reflects the controlled and symmetric nature of the laboratory setup. This agreement is expected for fibers placed at comparable axial locations around the pipe. However, we cannot generalize this observation to assert that ICVs of similar design in the field will necessarily share equivalent model coefficients. Instead, our results indicate that, in this particular experiment, two similarly deployed straight fibers yielded comparable responses.

Nevertheless, the results suggest a possible direction for future field application. If multiple ICVs share comparable hardware geometry and consistent DAS installation layouts, then it may be possible—pending field validation—to use a shared set of coefficients in a global model. In this scenario, the total surface-measured flow rate could be related to the summed DAS frequency band amplitudes (FBAs) and valve opening areas across all ICVs, leading to a formulation such as:

(17) $$\sum_{j}Q_{j}=\sum_{i}\beta_{i}\sum_{j}FBA_{i,j}A_{j}^{0.6}$$

Here, $Q_{j}$ is the unknown flow rate through the j-th ICV, $FBA_{i,j}$ is the DAS amplitude in the i-th frequency band near the j-th ICV, and $A_{j}$ is the valve opening area. This equation is not proposed as a validated field model, but rather as a concept that may be explored in future work, contingent on field data and operational feasibility. A dedicated field study would be required to determine whether shared or zone-specific coefficients are appropriate, and whether assumptions such as additive ICV contributions hold in heterogeneous reservoirs.

From a spatial-resolution perspective, DAS measurements should not be interpreted as point measurements directly at the ICV. The acoustic and vibrational energy generated by localized restricted flow can propagate along the pipe and be recorded by nearby fiber sections. Therefore, the measured DAS response represents a combination of localized energy generation at the ICV and spatially averaged wave/vibration propagation along the pipe. In this experiment, the DAS gauge length was approximately 2.5 m and the channel spacing was approximately 1 m, which limits the ability to resolve fine-scale flow structures or highly localized turbulence directly at the ICV. As a result, the straight fiber sections may provide more stable predictive performance potentially because they capture a more spatially averaged and higher-SNR response, whereas the coiled sections near the ICV are more sensitive to local installation variability, structural vibration, and complex flow mixing at the restriction. Future work should systematically evaluate the effect of sampling distance, gauge length, fiber geometry, and distance from the ICV on the sensitivity of DAS measurements to localized ICV dynamics.

This field-level application leverages the generalizability of the power-law models and enables zonal flow rate estimation without requiring intrusive measurements. While assumptions such as independent ICV contributions and uniform hardware may introduce some approximation error, this framework offers a scalable, physics-informed approach that can be further refined using field calibration data or integrated with data assimilation techniques for improved accuracy.

Beyond flow-rate estimation, several limitations should be acknowledged. All experiments were conducted under controlled laboratory conditions designed to approximate horizontal well inflow. Real field systems include greater uncertainty in reservoir inflow behavior, fiber-to-pipe coupling, thermal effects, and unsteady multiphase flow. The influence of fiber geometry on signal sensitivity, particularly differences between straight and helically wrapped sections, remains only partially understood and warrants further investigation.

**Figure 18:**
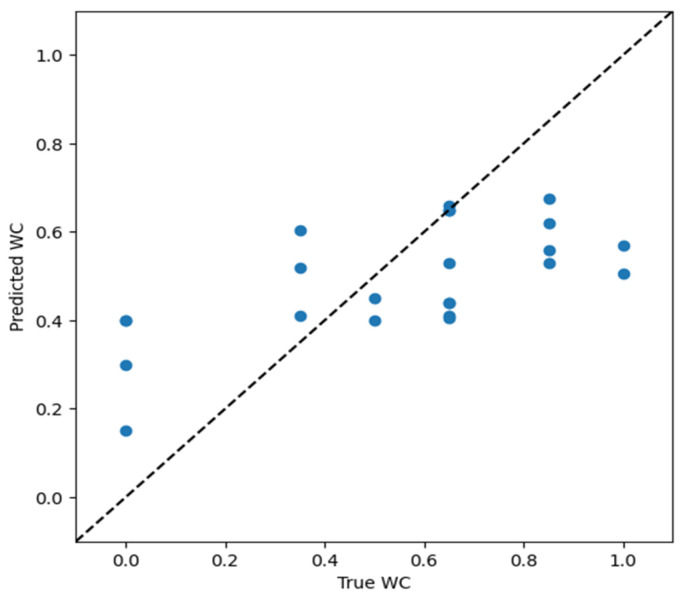
Cross plot of water cut estimation of test data from Straight 12 fiber section. Blue dots represent the estimated water cut values, and the dashed line represents the 0% mismatch line between the true and predicted water cuts.

For water cut estimation, extensive analyses were conducted; however, the results did not demonstrate satisfactory accuracy. To learn the nonlinear relationship between $C_{v}$ and water cut, a machine learning model was applied to correlate $C_{v}$, water cut, and valve opening. The $C_{v}$ coefficients, derived from experimental Pd, Q, and valve opening data from all tests, were used as model inputs, while water cut served as the output variable. Given the limited dataset size, a K-fold cross-validation was employed to enhance training robustness. In the cross plot of water cut estimation presented in Figure 18, the $R^{2}$ score of this estimation is only 0.3, indicating poor estimation performance, similar to MVA results using FBAs (Table 1). This outcome highlights the non-uniqueness and complexity of the $C_{v}$–WC relationship, where different combinations of WC and valve settings can yield similar $C_{v}$ values, making accurate water cut estimation challenging [24,26].

Future work will therefore focus on expanding the model inputs (e.g., phase-difference metrics, coherence analyses, and multi-band spectral relationships), incorporating physics-informed learning architectures, and conducting field trials integrating DAS, DTS, and pressure data. Such studies will be essential for assessing whether formulations like Equation (17) can become operational tools or require refinement for field-ready deployment.

## 5. Conclusions

A series of experiments were conducted to investigate the capability of Distributed Acoustic Sensing for characterizing oil–water two-phase flow under intelligent completion systems equipped with Inflow Control Valves. Using spectrogram analysis and polynomial regression, DAS frequency band amplitudes were shown to correlate strongly with flow rate and pressure drop across the ICV. A key contribution of this study is the development of a structured workflow for processing and analyzing DAS measurements under varying flow parameters, enabling extraction of stable and physically meaningful spectral attributes across different valve positions, and flow rates.

The regression model revealed a meaningful physical relationship: the FBA squared proportional to pressure drop and FBA proportional to flow velocity—highlighting DAS’s sensitivity to energy loss in the system. This power-law formulation represents a second major contribution of the work: it provides a quantitative link between DAS spectral amplitudes and fundamental flow behaviors near the ICV, capturing the empirical trends observed in the experiments. However, because the model coefficients are derived from controlled laboratory experiments, dedicated field validation is still required to evaluate their transferability to real well conditions with different completion geometries, fiber coupling, fluid properties, and operating conditions.

These findings provide a physical interpretation of DAS signal behavior that is generalizable to broader multiphase flow conditions. Most importantly, the power-law relationship offers insight into the physical mechanism of what the DAS fiber actually measures: local turbulence intensity and energy dissipation which is governed by flow physics rather than specific test-loop geometry. This physical grounding gives the model stronger potential for generalization and applicability to other facilities and operating conditions. This approach offers a promising direction for expanding DAS applications in production monitoring, especially in intelligent completions where traditional sensors are limited.

## Figures and Tables

**Table 1:** Multi-variance results for different flow parameters using DAS signal.

R^2^ Score	Coil 1	Coil 2	Coil 3	Straight 12	Straight 6
Pd	0.38	0.65	0.78	0.81	0.77
$Q_{annulus}$	0.3	0.46	0.47	0.43	0.46
$Q_{annulus}$(add valve open as input)	0.59	0.75	0.78	0.8	0.79
WC	0.35	0.24	0.36	0.32	0.32

**Table 2:** Coefficients of Pd measurement models.

	α_1_ (0–500 Hz)	α_2_ (500–1000 Hz)	α_3_ (1000–1500 Hz)	α_4_ (1500–2000 Hz)	α_5_ (2000–2500 Hz)	α_6_ (2500–3000 Hz)	Optimal n_1_	Best R^2^
Coil 1	−2.0 × 10^9^	2.6 × 10^9^	9.2 × 10^7^	−7.0 × 10^8^	6.3 × 10^8^	7.5 × 10^8^	2	0.51
Coil 2	−1.9 × 10^9^	1.9 × 10^9^	1.5 × 10^9^	2.3 × 10^9^	−1.6 × 10^9^	2.7 × 10^8^	2	0.68
Coil 3	−7.2 × 10^8^	1.9 × 10^9^	1.25 × 10^9^	2.4 × 10^9^	−5.4 × 10^8^	2.7 × 10^8^	2	0.76
Straight 12	3.1 × 10^8^	1.4 × 10^9^	−3.8 × 10^8^	−5.8 × 10^8^	1.3 × 10^9^	1.2 × 10^9^	2	0.80
Straight 6	1.6 × 10^8^	1.3 × 10^9^	−9.2 × 10^8^	−3.0 × 10^9^	4.7 × 10^9^	8.4 × 10^8^	2	0.79

**Table 3:** Coefficients of flow rate estimation models.

	β_1_ (0–500 Hz)	β_2_ (500–1000 Hz)	β_3_ (1000–1500 Hz)	β_4_ (1500–2000 Hz)	β_5_ (2000–2500 Hz)	β_6_ (2500–3000 Hz)
Coil 1	3.4 × 10^5^	−3.5 × 10^4^	2.7 × 10^4^	1.2 × 10^5^	−5.8 × 10^4^	5.7 × 10^4^
Coil 2	−1.0 × 10^5^	1.6 × 10^5^	1.8 × 10^5^	3.8 × 10^4^	−6.3 × 10^4^	5.1 × 10^4^
Coil 3	3.2 × 10^4^	8.2 × 10^4^	6.7 × 10^4^	−1.5 × 10^4^	−2.4 × 10^4^	1.1 × 10^4^
Straight 12	6.7 × 10^4^	5.6 × 10^4^	4.9 × 10^4^	1.2 × 10^5^	−1.1 × 10^5^	−4.7 × 10^4^
Straight 6	8.1 × 10^4^	6.7 × 10^4^	6.2 × 10^4^	7.1 × 10^4^	−1.0 × 10^5^	−7.1 × 10^4^

**Table 4:** Optimal fitting results of n_5_ for five water cut test groups.

Water Cut (%)	0	35	50	65	85
Optimal n5 value	−1.20	−1.22	−1.36	−1.25	−1.26

## Data Availability

The data presented in this study may be available on request from the corresponding author.
